# When Fertilization Is Not Enough: Maternal-Zygotic Transition as a Determinant of Embryo Competence in IVF

**DOI:** 10.3390/ijms27114787

**Published:** 2026-05-26

**Authors:** Charalampos Voros, Fotios Chatzinikolaou, Georgios Papadimas, Ali Can Gunes, Aristotelis-Marios Koulakmanidis, Ioannis Papapanagiotou, Athanasios Karpouzos, Diamantis Athanasiou, Kyriakos Bananis, Antonia Athanasiou, Aikaterini Athanasiou, Charalampos Tsimpoukelis, Maria Anastasia Daskalaki, Christina-Maria Trakatelli, Nikolaos Thomakos, Panagiotis Antsaklis, Dimitrios Loutradis, Georgios Daskalakis

**Affiliations:** 1 Department of Obstetrics and Gynecology, ‘Alexandra’ General Hospital, National and Kapodistrian University of Athens, 80 Vasilissis Sofias Avenue, 11528 Athens, Greece; aristoteliskoulak@gmail.com (A.-M.K.); thanoskarpouzosdr@hotmail.com (A.K.); tsimpoukelischa@gmail.com (C.T.); md181341@students.euc.ac.cy (M.A.D.); thomakir@hotmail.com (N.T.); panosant@gmail.com (P.A.);; 2 Laboratory of Forensic Medicine and Toxicology, School of Medicine, Aristotle University of Thessaloniki, 54124 Athens, Greece; 3 Athens Medical School, National and Kapodistrian University of Athens, 15772 Athens, Greece; dr.georgepapadimas@gmail.com (G.P.); gpapamd@hotmail.com (I.P.); diamathan16@gmail.com (D.A.); antoathan16@gmail.com (A.A.); diamathan17@gmail.com (A.A.); loutradi@otenet.gr (D.L.); 4 Department of Obstetrics and Gynecology, Faculty of Medicine, Hacettepe University, Ankara 06100, Turkey; acgunes@hacettepe.edu.tr; 5 King’s College Hospitals NHS Foundation Trust, London SE5 9RS, UK; kyriakos.bananis@nhs.net; 6 3rd Department of Internal Medicine, Aristotle University, 54124 Thessaloniki, Greece; ctrak@auth.gr

**Keywords:** maternal-zygotic transition, zygotic genome activation, oocyte competence, embryo development, embryo arrest, in vitro fertilization, maternal mRNA clearance, translational control, early embryogenesis

## Abstract

A significant concern with IVF is that many embryos fail to develop despite proper fertilization. This gap indicates that factors outside sperm-oocyte fusion influence developmental competency. The maternal-zygotic transition (MZT) is a crucial developmental phase during which control shifts from maternally inherited transcripts to activation of the embryonic genome. During the early stages following fertilization, the embryo depends only on maternal mRNA and proteins amassed throughout oogenesis. For successful development, these transcripts must be expeditiously removed with the concurrent genome activation. Any disruption, whether due to inadequate maternal mRNA degradation, aberrant translational control, or delayed genome activation, has been associated with premature developmental stoppage and diminished blastocyst formation. Principal regulators, such as BTG4, CPEB1, DAZL, and components of the translational machinery, govern this modification and seem to be affected by the quality of the oocyte and the age of the mother. Increasing evidence suggests that disruption of MZT may account for instances of suboptimal embryo development that conventional assessment techniques cannot elucidate. MZT offers a biological framework for assessing embryo competency beyond simple appearance. If scientists had a deeper understanding of this process, they might identify molecular markers and enhance the selection of embryos in IVF.

## 1. Introduction

In vitro fertilization has transformed infertility therapy. Early embryonic developmental failure remains a considerable barrier, even in cycles with normal fertilization. The presence of two pronuclei and early cleavage does not reliably signify progression to the blastocyst stage or successful implantation [1]. In normal embryology practice, a substantial proportion of embryos undergo arrest in the early days post-fertilization, without an identifiable morphological explanation. Early embryogenesis begins in a transcriptionally inactive condition [2]. In the early phases post-fertilization, development mostly relies on maternal reserves accumulated during oogenesis, including messenger RNAs, proteins, organelles, and regulatory elements that support cleavage, chromatin remodelling, and metabolic adaptation. To facilitate ongoing development, maternal control must be systematically relinquished, followed by the activation of embryonic transcription. The maternal-zygotic transition (MZT) is a critical juncture in preimplantation development that significantly influences the embryo’s developmental potential and development potential [3].

MZT is defined by two tightly synchronised processes: the degradation of maternally transmitted transcripts and the activation of the zygotic genome. Rather than a passive transmission of developmental control, the transition entails a comprehensive reprogramming of both the cytoplasmic and nuclear compartments. Chromatin architecture is gradually reorganised to enable the activation of the embryonic genome, while maternal RNAs are selectively degraded, stabilised, or translated in accordance with developmental timing [2,4]. Any interruption in this process may impede cleavage advancement, delay developmental kinetics, threaten blastocyst formation, and reduce reproductive capacity.

Increasing data indicates that the elimination of maternal RNA is a meticulously regulated process that directly influences development. Transcript degradation starts during oocyte maturation and continues after fertilization via temporally coordinated maternal and zygotic mechanisms [2,5]. The destiny of transcripts is governed by poly(A) tail dynamics, RNA-binding proteins, translational regulators, and deadenylation complexes. A subset of maternal mRNAs is accessible for selective translation during early development [2,6]. Recent research has expanded this approach by identifying autophagy-related components, such as LC3B, as active participants in maternal mRNA degradation, therefore linking cytoplasmic recycling mechanisms with developmental competence [7].

Human findings validate the clinical importance of MZT. Sha and colleagues clarified that maternal mRNA clearance in human embryos follows distinct M-decay and Z-decay routes, with impairments in either pathway strongly associated with developmental arrest during assisted reproduction [5]. Embryos arrested at the zygote or 8-cell stage demonstrate aberrant retention of maternal transcripts and reduced expression of factors associated with RNA destabilisation and zygotic activation. These results place faulty MZT at the center of a clinically recognisable pattern characterised by normal fertilisation followed by failed embryonic development [8].

Zygotic genome activation (ZGA), the second most critical component of MZT, is essential for the continuation of development. Embryonic transcription must begin within a favourable nuclear environment defined by chromatin remodelling, epigenetic reconfiguration, and precise transcriptional control [9]. Recent study has highlighted the complexity of ZGA regulation and proposed multiple regulatory factors that promote transcriptional start or particular gene activation at different stages [10]. Single-cell transcriptomic investigations of human embryos have shown stage-dependent changes in gene expression, demonstrating a highly organised developmental program rather than a gradual or generic transcriptional activation [11,12,13]. Comparisons across species confirm the maintenance of the overall structure, while also emphasizing notable temporal and regulatory differences.

The growing interest in MZT reflects its biological importance and its potential relevance to reproductive medicine. Impaired cytoplasmic inheritance, defective maternal transcript regulation, altered translational control, or incomplete zygotic activation have been proposed as potential contributors to suboptimal embryo development, developmental arrest, low blastocyst yield, and some forms of unexplained IVF failure. This perspective may be particularly relevant in clinical settings where embryo assessment still relies predominantly on morphological and morphokinetic evaluation rather than direct molecular characterization [14].

This narrative review examines the maternal-zygotic transition as a determinant of early embryo competency in IVF. The emphasis is on maternal mRNA degradation, translational control, zygotic genome activation, and the molecular processes that enable the shift from oocyte-mediated to embryo-mediated development. Human evidence is emphasised where accessible, although specific experimental research is included to clarify systems that are difficult to examine directly in human embryos. To provide a structured overview of the molecular processes governing the maternal-zygotic transition, the key regulatory events are summarised in Table 1.

Table 1 shows the main molecular processes that control the MZT. Each mechanism enables the coordinated transition from maternal to embryonic regulation. Any disruption may compromise embryo viability and lead to premature developmental cessation.

**Figure 1:**
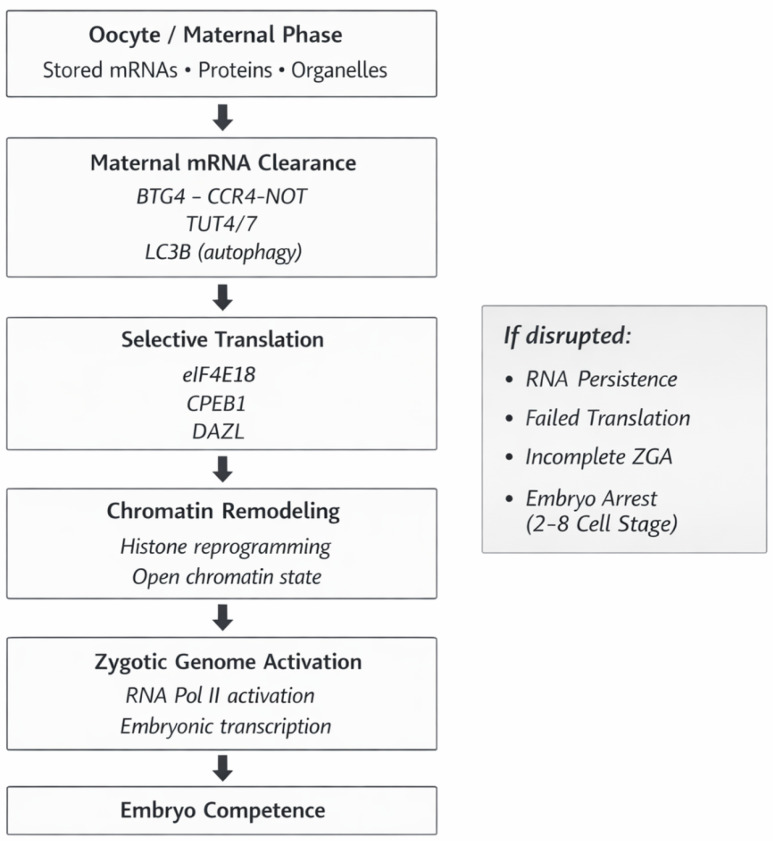
Molecular sequence of the maternal-zygotic transition in early human embryogenesis.

The sequence of molecular events underlying the maternal-zygotic transition is illustrated in Figure 1.

Maternal transcripts and proteins facilitate the initial development of the embryo. Gradually getting rid of maternal mRNAs and selectively activating key factors for translation prepare the cytoplasmic and nuclear environments for chromatin remodelling and the activation of the embryonic genome. Successful coordination of these events leads to the establishment of embryonic transcriptional autonomy and developmental competence. If something goes wrong at any point during this process, it could cause the embryo to stop growing early, usually at the cleavage stage.

Prior studies mostly focused on the developmental biology of maternal-zygotic transition, whereas the current study highlights its translational significance in human IVF and embryo viability. Our research elucidates the correlation between poor transcript clearance, compromised genome activation, developmental stasis, and unexplained IVF failure, integrating current discoveries from human embryology, single-cell transcriptomics, and innovative molecular techniques pertinent to assisted reproduction.

## 2. Early Human Embryogenesis: The Maternal Phase

Following fertilization, the human embryo undergoes a period of transcriptional quiet, during which developmental progression is only supported by molecular components accumulated during oogenesis [15]. At this stage, the zygote is incapable of transcribing its own DNA and requires a preformed cytoplasmic program composed of maternal messenger RNAs, storage proteins, ribosomes, mitochondria, and regulatory complexes [4]. The inherited molecular reservoir, rather than novel gene expression, sustains cleavage divisions, cytoskeletal architecture, chromatin remodelling, and initial metabolic activity. Pronuclear formation, DNA replication, and the first mitotic divisions occur before substantial embryonic transcription begins. This highlights the extent to which early development is predetermined during oocyte maturation. Thus, any discrepancy in the amount or quality of these maternal elements may have direct effects on developmental advancement before the activation of the embryonic genome [16,17].

At this stage, maternal transcripts constitute the primary regulatory layer. During oocyte development, many mRNAs are synthesized, although only a subset is retained in a translationally inactive state [18]. Post-fertilization, they are activated in a stage-specific manner. The regulation of translation, rather than transcription, determines the quantity of accessible protein. Post-transcriptional regulation is therefore the essential aspect of early developmental control [19,20].

Translational activation is highly selective and evolves over time within the maternal transcriptome. A subset of mRNAs is rapidly recruited to ribosomes immediately after fertilization. These mRNAs encode proteins essential for chromatin remodelling, the cell cycle, and preparation for zygotic genome activation [20]. Ribosome profiling studies have shown a significant change in translational activity between the metaphase II egg and the zygote. This indicates that protein production is actively reconfigured from the onset of embryogenesis, rather than being static [21]. To establish the prerequisites for subsequent genome activation, certain maternal variables influencing chromatin accessibility and transcriptional competency must be translated. Disruption of the selective translation program, whether due to ribosome recruitment issues or improper control of initiation factors, would prevent the embryo from achieving transcriptional autonomy [22].

At this juncture, the architecture of the cytoplasm introduces an additional layer of regulation. Mitochondria inherited from the oocyte serve as the only source of ATP, enabling cleavage divisions, biosynthetic activities, and the maintenance of cellular homeostasis [23]. The quantity, location, and functional integrity of mitochondria influence energy availability and redox balance, therefore impacting translation efficiency and protein folding. Mitochondrial dysfunction may lead to increased production of reactive oxygen species, alter metabolic pathways, and impede the activation of critical developmental processes. Cytoskeletal elements simultaneously facilitate spindle positioning, chromosomal separation, and spatial organization of blastomeres. For symmetric cell division and the maintenance of developmental potential, organelles and macromolecular complexes must be accurately positioned inside the ooplasm [24].

Despite the absence of transcription, the nuclear compartment undergoes significant reprogramming. Subsequent to fertilization, paternal chromatin rapidly undergoes decondensation, with protamines being substituted by histones. This enables the formation of a transcriptionally permissive structure. Maternal chromatin undergoes remodelling, which entails the repositioning of histones and nucleosomes [25]. Epigenetic modifications inherited from gametes are selectively removed or altered, facilitating the establishment of a totipotent state. Alterations in chromatin accessibility start prior to the evident activation of transcription [26]. This indicates that occurrences before to and during zygotic genome activation transpire inside the nucleus. Thus, coordination between cytoplasmic signals and nuclear remodelling is crucial, as incorrect chromatin reprogramming may impede or obstruct the initiation of embryonic transcription.

The quality of the oocyte, established during folliculogenesis, directly influences the health of the maternal phase. The interaction between the oocyte and the surrounding granulosa cells regulates the accumulation of transcripts, proteins, and organelles essential for subsequent embryonic development [27]. Metabolic abnormalities, oxidative stress, or hormonal imbalances during oocyte maturation may alter the composition of maternal reserves, leading to compromised cytoplasmic maturation. These alterations may not directly influence fertilization; nonetheless, they might diminish the efficacy of translational regulation, mitochondrial function, and chromatin remodelling in the early embryo [28]. Clinical findings of normal fertilization followed by early developmental stoppage suggest that abnormalities originate from oocyte-derived molecular programming rather than sperm-related variables [18,29].

The first phases of embryogenesis are mostly regulated by maternally inherited factors. However, there is growing evidence indicating that paternal contributions may play a role in the maternal-zygotic transition. Spermatozoa transport the paternal genome along with short non-coding RNAs, messenger RNAs, proteins, and epigenetic information, which may have a role in chromatin remodelling, transcript control, and early embryonic signalling pathways. Research indicates that sperm-derived RNAs may influence zygotic gene expression and developmental timing, whereas paternal epigenetic modifications may play a role in the formation of early chromatin structure. Paternal factors contribute less in comparison to the maternal regulatory reservoir. Their interaction with oocyte-derived molecular pathways may affect embryonic competence and developmental advancement.

Clinically, these alterations may have special importance for women of advanced maternal age, those with reduced ovarian reserve, metabolic dysfunction, or those experiencing repeated IVF cycles characterised by normal fertilization but suboptimal blastocyst development or recurrent early embryonic arrest. These clinical traits may result from impaired programming of oocyte-derived molecules and inadequate maternal assistance throughout the pre-ZGA developing period.

## 3. Maternal mRNA Clearance

An essential component of early embryogenesis is the progressive degradation of maternal transcripts. To achieve embryonic control, it is essential to promptly and selectively remove the accumulated messenger RNAs from oocyte development. The persistent presence of maternal transcripts beyond their functional period disrupts gene expression pathways critical for future development, while premature degradation may deprive the embryo of necessary regulatory proteins [28]. Transcript clearance occurs in a highly regulated manner, rather than occurring passively. In addition to basic turnover, the elimination of maternal RNA is a crucial aspect of reprogramming that alters the molecular architecture of the early embryo [30]. Eliminating transcripts that previously facilitated oocyte maturation paves the way for a novel transcriptional program and prevents the retention of regulatory signals that might disrupt the timing of early development. The efficacy and accuracy of this process affect both the commencement of embryonic transcription and the regulation of the cell cycle, metabolism, and lineage determination [5].

The degradation of maternal mRNA starts during the latter phases of egg maturation and persists post-fertilization via independent but convergent processes. Two main pathways of decay have been identified: maternal-driven decay (M-decay), which starts before the activation of the embryonic genome, and zygotic-driven decay (Z-decay), which relies on transcriptional activity subsequent to genome activation [31]. M-decay targets certain transcripts for early degradation to facilitate the continuation of the cell cycle and the reorganisation of the cytoplasm. Z-decay eliminates transcripts that persist until subsequent cleavage stages. The coordination of these routes facilitates a gradual transition from maternal control, therefore preventing sudden interruptions in developmental processes [32]. The temporal disparity between M-decay and Z-decay enables the embryo to continue essential processes while preparing for transcriptional activation. Disruption of either route alters the equilibrium between preserved and degraded transcripts, resulting in asynchronous development and coordination issues in cytoplasmic and nuclear activities. Data from human embryos suggest that different developmental arrest phenotypes may signify failures at separate phases of transcript clearance, underscoring the functional specificity of each route [33].

Numerous post-transcriptional mechanisms collaborate effectively to regulate the degradation of maternal mRNA at the molecular level. Deadenylation, performed by complexes such as CCR4-NOT, truncates poly(A) tails and designates transcripts for degradation. RNA-binding proteins identify sequence motifs in target mRNAs and regulate their stability or degradation [34]. They often work with enzymatic complexes that help remove transcripts. Alterations to the 3′ untranslated region, such as uridylation, enhance transcript instability and increase vulnerability to degradation. Simultaneously, certain maternal factors, such as BTG4, function as adaptors that connect RNA-binding proteins to the degradation machinery, enhancing the selectivity of the process [35]. Regulation is heterogeneous across the transcriptome; some subsets of mRNAs are designated for distinct developmental phases, while others are retained for ongoing translation. This selection ensures the retention of proteins essential for early cleavage while promptly eliminating transcripts incompatible with subsequent developmental stages. Alterations to this regulatory network may result in transcripts persisting excessively or degrading prematurely, both of which would adversely affect embryonic development [18].

The efficacy of translation is intricately linked to the stability of transcripts. This renders the link between RNA turnover and protein production very dynamic. Ribosomes and translational complexes often safeguard actively translated mRNAs against degradation [36]. Conversely, transcripts that are subject to translational repression are more prone to degradation. Variations in the length of the poly(A) tail influence the efficiency of RNA translation and its stability. This connects cytoplasmic polyadenylation with the control of transcript fate [37]. Throughout the transition from egg to zygote, the translational landscape undergoes significant remodelling, marked by the selective recruitment of maternal transcripts to ribosomes and the concurrent destruction of superfluous transcripts. This kind of remodelling ensures that protein synthesis aligns with the requirements of each developmental stage. If translation and degradation are improperly executed, it may result in the accumulation of non-functional transcripts or insufficient production of proteins essential for chromatin remodelling, cell cycle progression, and genome activation. Consequently, translational misregulation constitutes a significant vulnerability throughout the first phases of embryogenesis [38].

Recent discoveries have broadened the processes of maternal mRNA clearance by including cytoplasmic degradation pathways typically linked to protein turnover. Components associated with autophagy have been shown to be directly implicated in RNA degradation. Proteins such as LC3B function as RNA-binding factors that facilitate the degradation of certain maternal transcripts [7]. While these systems have mostly been studied in embryonic models of zebrafish and mice, little is known about their exact function and regulation dynamics in human embryos. These results indicate that autophagy plays a role in organelle recycling and the regulation of RNA dynamics in the early embryo. Inhibiting autophagy impedes transcript clearance, disrupts zygotic genome activation, and halts development. This indicates that efficient cytoplasmic recycling is essential for a seamless shift to embryonic regulation [7]. Incorporating autophagic activity into conventional RNA decay pathways enhances control over metabolic states and cellular homeostasis, which is associated with transcript turnover. That relationship may elucidate the interconnections among metabolic disorders, cytoplasmic stress, and suboptimal embryo development [39].

Human findings provide direct evidence connecting reduced maternal mRNA clearance to obstructed embryo development. Studies on preimplantation embryos have shown that the aberrant retention of maternal transcripts often happens in embryos that halt development at the zygote or early cleavage stages [5]. Issues with the activation of the embryonic genome are associated with reduced amounts of critical RNA decay regulators, such as those involved in deadenylation and transcript destabilisation. Reports indicate various forms of disturbance. Early arrest correlates with issues related to M-decay, while later arrest correlates with issues pertaining to Z-decay [40]. The accumulation of maternal transcripts beyond the designated developmental period creates a molecular milieu that hinders transcriptional activation and synchronised developmental advancement. These results provide a definitive mechanistic elucidation for instances of normal fertilization followed by premature developmental cessation in IVF, underscoring the therapeutic significance of transcript clearance pathways [5].

Precision in timing is crucial for effective progression through this phase. For development to occur in a coordinated manner, transcript clearance must coincide with translational activation, chromatin remodelling, and alterations in the cell cycle. If maternal transcripts persist excessively, they may inhibit transcriptional activation by retaining inappropriate regulatory signals or competing with freshly synthesised RNAs [41]. Conversely, if the transcripts required for early cleavage degrade prematurely, it may impair critical cellular activities and hinder development. To sustain this balance, several interconnected pathways need precise control, each affecting the overall timing and effectiveness of transcript turnover. Minor alterations in timing or dimensions may significantly impact embryo viability, underscoring the critical importance of developmental events occurring within a brief timeframe [42].

The degradation of maternal mRNA signifies more than only a preliminary stage before genome activation. The control of transcript turnover establishes the molecular milieu for the initiation of embryonic transcription and influences the progression of early development. Errors at this stage may go undetected by standard morphological evaluations. However, they might influence whether the embryo advances to the next developmental phase or terminates prematurely. Recognising transcript dynamics as a crucial factor in embryo competency adds a molecular aspect to embryo assessment that goes beyond morphology and morphokinetics. Incorporating these findings into clinical practice may enhance the prediction of developmental potential and refine embryo selection procedures in assisted reproduction.

## 4. Zygotic Genome Activation

Zygotic genome activation (ZGA) denotes the commencement of transcription from the embryonic genome, crucial for continued development. Transcriptional activity in human embryos evolves gradually, including both minor and large stages of zygotic genome activation. A restricted initial wave of transcriptional activity commences during the pronuclear and early cleavage stages, lasting until roughly the 4-cell stage, while the principal wave of zygotic gene activation predominantly transpires at the 8-cell stage, linked to extensive activation of embryonic transcriptional programs [4]. To facilitate cleavage, a transcriptionally competent genome must be established to enable cell cycle progression, metabolic regulation, and initial lineage determinations. Prior to that, transcriptional silence is maintained despite ongoing chromatin remodelling. This indicates that genome activation requires both structural and regulatory preparedness [43]. The shift to transcriptional autonomy is gradual, including a progressive change in regulatory supremacy, where maternal transcripts continue to contribute while embryonic transcription increases. The timing of that transition seems to be rigid, since even little delays in the initiation of transcription might result in asynchronous development, complicating the collaborative function of blastomeres. Variations in the timing or intensity of genome activation have been associated with disparities in developmental potential [44].

Despite the fact that the general biological framework of zygotic genome activation is conserved across mammals, there are significant distinctions between murine and human embryogenesis. The principal phase of activation in human embryos occurs later, primarily between the 4-cell and 8-cell stages, whereas in rodents, substantial zygotic genome activation occurs predominantly at the 2-cell stage [9,10]. In addition, human embryos exhibit distinct transcriptional and epigenetic dynamics in comparison to murine systems, and they are dependent on maternal transcripts for an extended period. The developmental timing and regulatory context of the pathways implicated in RNA decay, chromatin remodelling, and transcriptional licensing appear to be evolutionarily conserved, but they may differ significantly between species. Therefore, mechanistic discoveries from animal models are highly informative for comprehending MZT biology. However, direct extrapolation to human IVF should be approached with caution and substantiated by human embryo data whenever feasible.

The chromatin structure undergoes significant alterations as cells attain transcriptional competence. Paternal chromatin, originally linked to protamines, is rapidly reorganised with histones after fertilization, leading to a more accessible chromatin structure that facilitates future transcriptional activity [45]. Maternal chromatin experiences gradual remodelling, which entails the repositioning of nucleosomes and the alteration of histone modifications associated with gene activation or repression. The trimethylation of histone H3 at lysine 4 (H3K4me3) and the reduction in repressive marks, such as H3K9me3, promote the formation of transcriptionally permissive areas [46]. Substantial modifications in chromatin accessibility transpire before detectable transcription, indicating that genome-wide reorganization establishes the structural basis essential for ZGA. At this stage, three-dimensional chromatin architecture begins to form. This encompasses the formation of chromatin loops and topologically linked regions that influence gene regulation. If chromatin is not adequately organized, transcription factors and RNA polymerase II may be unable to access regulatory areas [47].

For the transcriptional machinery to function, RNA polymerase II complexes must assemble in conjunction with their corresponding transcription factors in a coordinated manner. The phosphorylation of the C-terminal domain of RNA polymerase II indicates the transition from transcription start to elongation, promoting the effective synthesis of RNA transcripts [48]. Initial embryonic transcription is characterised by brief, uncomplicated transcripts that evolve into longer and more complex forms as development progresses and transcriptional capacity expands. The initial transcriptional output seems sufficient to initiate regulatory cascades that enhance gene expression and maintain the stability of the embryonic program. During the cleavage phases of embryonic development, the efficiency of transcriptional elongation, RNA processing, and splicing improves. Improper assembly of transcriptional complexes or failure to maintain elongation may result in incomplete or abortive transcription, adversely affecting subsequent developmental processes [49].

Regulation of ZGA includes both permissive and instructive components that operate at several levels of gene expression. Chromatin remodelers, histone-modifying enzymes, and nuclear proteins that regulate chromatin accessibility and transcriptional preparedness facilitate the initiation of transcription. Simultaneously, transcription factors that are unique to certain sequences activate clusters of genes essential for cleavage progression and early lineage determination [9]. The connections among these regulatory layers ensure that transcription initiates at the appropriate time and occurs exclusively in the correct regions of the genome. Recent data has identified many regulators that facilitate transcriptional activation or govern gene-specific expression patterns. This indicates that control systems are structured in a hierarchical manner [50]. Among the transcriptional regulators involved in early human zygotic activation, DUX4 has emerged as a potential pioneer transcription factor that may initiate gene expression programs at the cleavage stage and activate repetitive motifs linked to early embryonic transcriptional remodelling. The ephemeral and highly constrained expression pattern of DUX4 during early human embryogenesis indicates its potential involvement in starting the shift towards embryonic transcriptional independence. Members of the OBOX family of homeobox transcription factors have been associated with the control of early embryonic gene expression and developmental competence. These variables are proposed to participate in transcriptional activation networks linked to cleavage-stage progression and the commencement of early developmental programs. Their exact regulatory connections in human embryos remain incompletely known. Misregulation at any level may lead to erroneous gene activation, failure to initiate essential transcriptional processes, or activation of genes at unsuitable developmental stages. Such issues disrupt the typical sequence of embryonic processes and may result in embryos that cease growth or develop abnormally [51].

Epigenetic reprogramming is crucial to zygotic genome activation and facilitates the transcriptional activation of the genome. In the first stages of development, DNA demethylation occurs across the whole genome. The dynamics of paternal and maternal genomes vary, indicating the use of distinct regulatory mechanisms [52]. Passive demethylation via replication-dependent dilution and active enzymatic processes reduces methylation levels, hence enhancing accessibility to hitherto suppressed regulatory areas. Simultaneously, maintaining methylation at imprinted loci for particular genes preserves the expression of parent-of-origin-specific genes, therefore preserving crucial epigenetic information [53]. The interaction between DNA methylation dynamics and histone modifications ensures that gene activation occurs in a controlled and developmentally appropriate manner. Interference with epigenetic reprogramming may lead to aberrant transcriptional profiles, altered chromatin states, and impeded developmental progress. Epigenetic instability at this period is associated with lasting impacts on embryo viability and post-implantation development [54].

RNA metabolism remains intricately associated with transcriptional activation and is crucial for delineating the nascent embryonic transcriptome. Prior to the export of freshly synthesized transcripts to the cytoplasm for translation into functional proteins, they must undergo processing, capping, splicing, and polyadenylation [55]. The interplay between emerging transcription and remaining maternal RNA reserves creates a transitional regulatory framework in which both transcript sources coexist and influence gene expression trends. The efficient turnover of maternal mRNAs facilitates embryonic transcriptional dominance, whereas delayed clearance may interfere with the regulatory networks critical for ZGA. RNA-binding proteins and post-transcriptional modifiers regulate transcript stability, localization, and translation, hence influencing gene expression even post-transcription. Coordination between transcription and RNA processing ensures that freshly synthesized transcripts efficiently promote developmental advancement [4].

Single-cell transcriptomic investigations have shown that gene expression throughout early human development follows a meticulously planned pattern, rather than a gradual or random progression. Every developmental stage has a distinct transcriptional program that activates certain gene groups regulating the cell cycle, metabolism, chromatin architecture, and cellular differentiation [56]. Stage-specific transcriptional modules manifest in a predetermined sequence across time, indicating that developmental timing is highly regulated. Alterations to that sequence, such as the delayed activation or complete inactivation of certain gene modules, are associated with complications in embryonic development and diminished developmental competence [57]. Arrested embryos often have transcriptional patterns that fail to transfer effectively from maternal to embryonic regulation, suggesting that deficits in zygotic gene activation are a primary cause of early developmental failure. The integration of transcriptome data with developmental outcomes has provided significant insights into the molecular determinants influencing embryo viability [58].

ZGA is a meticulously regulated molecular alteration that integrates chromatin remodelling, transcriptional activation, epigenetic reprogramming, and RNA processing into a cohesive developmental program. For effective operation, activation must occur timely and in harmony across several regulatory levels, each facilitating transcriptional autonomy. Insufficient or irregular activation interferes with subsequent developmental pathways, leading to hindered cleavage advancement, abnormal blastocyst formation, and reduced embryo survival. In assisted reproduction, the failure of ZGA clarifies the processes behind embryos that attain normal fertilization but do not progress beyond the earliest cleavage phases. Recognising ZGA as a crucial element in embryo competency highlights the need of molecular analysis in conjunction with conventional morphological examination [59].

## 5. Molecular Regulators of the Maternal-Zygotic Transition

The maternal-zygotic transition is governed by a network of closely interconnected regulatory elements rather than a single switch. RNA-binding proteins, deadenylation factors, translational regulators, chromatin remodelers, and nuclear gatekeepers collaborate to eliminate maternal transcripts, preferentially translate stored mRNAs, facilitate chromatin accessibility, and initiate embryonic transcription [41]. The operational integrity of that network determines whether the embryo progresses from maternally supported cleavage to independent development or ceases during the early stages. Molecular regulation is thereby dispersed across cytoplasmic and nuclear compartments, with ongoing interplay between RNA destiny and chromatin configuration [60]. A fundamental biological notion emerging from recent studies is that the quantity of transcripts alone does not account for an organism’s developmental efficacy. The timing of translation, RNA accessibility, recruitment of degradation machinery, and the creation of a conducive transcriptional environment are all equally significant. Developmental failure may arise from the lack of crucial molecules, the retention of factors that should have been removed, or the postponed activation of proteins required just during a certain developmental phase. This degree of temporal precision renders MZT analogous to a checkpoint [61].

BTG4 is a prominent regulator of maternal mRNA degradation. It is crucial in eliminating transcripts post-fertilization. BTG4 functions as a link between maternal transcripts and the CCR4-NOT deadenylase complex. This accelerates the truncation of poly(A) tails and the destabilisation of transcripts [62]. Deadenylation not only degrades RNA but also alters the efficiency of translation and the availability of maternal proteins during the first stages of cleavage. In mammalian systems, compromised BTG4 activity results in the aberrant retention of maternal RNAs, hindered developmental progression, and the failure to establish an appropriate molecular milieu for genome activation. BTG4’s biological importance lies in its capacity to modulate the selectivity and timing of transcript degradation [62]. In the absence of this regulatory mechanism, transcripts that facilitate oocyte maturation remain excessively elevated post-fertilization, complicating the transition to embryonic control. Human studies demonstrating reduced expression of BTG4-associated decay machinery in stopped embryos support the notion that inadequate deadenylation is not just an experimental phenomenon but a therapeutically relevant cause of developmental failure.

TUT4 and TUT7 are further implicated in regulating the stability of maternal RNA. They facilitate 3′ oligouridylation, hence enhancing the degradation of transcripts designated for elimination. Uridylation functions as a post-transcriptional signal that marks certain RNAs for destruction, often after the preceding truncation of the poly(A) tail. Consequently, RNA turnover emerges as a multifaceted process, including deadenylation, uridylation, and the simultaneous or sequential recruitment of exonucleolytic machinery [63]. This degree of complexity enables the embryo to distinguish between transcripts that must be promptly eliminated and those that may remain functioning for an extended period. A similar regulatory framework pertains to programs associated with YAP1–TEAD4, which have been correlated with human MZT via associations with pathways relevant to Z-decay. Rather than only acting as downstream transcriptional factors, such regulators may play a role in coordinating the shift from maternal clearance to embryonic activation. Their importance arises from the rarity of isolated faults in transcript removal. The disruption of a single post-transcriptional phase often results in the compromised activation of embryonic transcriptional modules. RNA degradation and transcriptional competence operate as mechanistically interrelated processes rather than independent events [64].

Regulation also functions at the level of translational control, whereby stored maternal transcripts are used for protein synthesis exclusively at certain temporal intervals. Early embryos cannot rely on fresh transcription to fulfil their immediate developmental requirements; hence, the translation of existing mRNAs becomes the primary mechanism for rapid molecular adaptation [18]. The translational apparatus, particularly cap-binding proteins and initiation complexes, is crucial in determining whether transcripts are directed to ribosomes or remain inactive. Investigations into mammalian embryos have highlighted the importance of eIF4E1B, a germ cell-abundant cap-binding protein that plays a crucial role in the selective translation of maternal mRNAs necessary for ZGA [65]. The functional significance of this element lies in its specificity: not all stored transcripts should be translated post-fertilization, and indiscriminate ribosome loading would be as harmful as failed translation. Selective translational activation allows the embryo to synthesise proteins crucial for chromatin remodelling, cell cycle progression, and transcription initiation, without the need of fresh RNA synthesis. Failure at that stage produces a notably adverse phenotype, since the transcript may remain but the necessary protein does not accumulate [66].

CPEB1 and the cytoplasmic polyadenylation-dependent regulation of maternal mRNAs are a component of a broader framework of translational control. CPEB1 recognises cytoplasmic polyadenylation regions in target transcripts and regulates the elongation of poly(A) tails, influencing both translational activation and transcript stability [67]. This kind of regulation is crucial throughout the transition from egg to embryo, since the dynamics of the poly(A) tail influence the fate of mRNA, determining whether it is stored, translated, or targeted for degradation. CPEB1 is crucial to both translation and clearance processes. Interference with CPEB1-regulated pathways may alter the rates of protein synthesis crucial for meiotic completion, fertilization-associated remodelling, and the readiness for embryonic genome activation. Additional RNA-binding proteins that recognise sequence features in 3′ untranslated regions and determine the fate of the transcript based on temporal need may also function as intermediaries [68]. The maternal transcriptome should be seen not as a static repository, but as a hierarchically regulated substrate in which each RNA type is continually assessed for storage, use, or elimination. This concept elucidates why embryos may cease development despite normal transcript abundance profiles: biological failure may occur due to improper management of RNA destiny, rather than just due to depletion of transcripts [69].

DAZL, an RNA-binding protein historically associated with germ cell development, is becoming recognised as a significant regulator in post-fertilization regulation. DAZL interacts with certain maternal transcripts and alters their translational processes. This is particularly applicable to aspects related to meiotic competence, RNA metabolism, and developmental advancement [70]. Although the majority of mechanistic details derive from non-human systems, DAZL illustrates a broader concept pertinent to MZT: lineage-restricted RNA-binding proteins regulate access to the maternal message pool to establish developmental competence. These proteins not only stabilise RNAs but also assist in identifying which biological programs remain active during the brief interval between the initiation and cessation of embryonic transcription [71]. Thus, DAZL and analogous factors function as molecular filters that maintain developmental order. The loss of this regulation may alter the kind of proteins accessible during cleavage and may hinder the zygote’s maturation in the cytoplasm and diminish the efficacy of the processes required for ZGA. The early embryo may depend on a consortium of RNA-binding proteins, since several RBPs exhibit stage-specific expression and target selectivity, rather than relying only on a singular primary regulator.

There is increased interest in YTHDF2 and the impact of RNA modification-dependent regulation on developmental transitions. YTHDF2 recognises N6-methyladenosine (m6A), a post-transcriptional modification of RNA that influences the stability, localisation, and translation of mRNA [72]. YTHDF2 promotes selective RNA degradation by identifying methylated transcripts, assisting in the removal of maternal signals that should not endure into later stages. The biological importance of m6A-mediated regulation lies in the enhanced specificity it provides [73]. Sequence patterns alone may not entirely determine transcript destiny; chemical modifications of RNA provide a dynamic coding that marks transcripts for retention or destruction according to developmental needs. This method is particularly advantageous in the context of MZT since it enables rapid transcriptome remodelling without the need for additional transcription. Disruption of m6A readers or writers may lead to a phenotype characterised by transcript persistence, prolonged transition, and the failure of embryonic activation. RNA alterations complicate MZT control. They provide a more comprehensive understanding of how embryos may rapidly and selectively alter their transcripts [74].

Chromatin remodelling factors originating from maternal mRNAs are a crucial class of regulators, since embryonic genome activation requires an appropriate nuclear environment. Research using ribosome profiling has identified maternal transcripts with significantly increased translation after fertilization, and their corresponding protein products are crucial for chromatin reprogramming and the commencement of transcription [6]. Among these factors, SMARCD2 is recognised as crucial for chromatin remodelling, whereas Cyclin T2 is linked to the promotion of ZGA-related transcriptional processes. The functional importance of these proteins goes beyond just opening chromatin; it includes the reconfiguration of promoter accessibility, enhancer effectiveness, and transcriptional elongation capacity [75]. Maternal translation prepares the nucleus for embryonic transcription before the full activation of the embryonic genome. The failure of this preliminary translation separates cytoplasmic preparedness from nuclear competence. Despite adequate degradation of maternal transcripts, genome activation may not occur if chromatin remains inaccessible or if RNA polymerase II is dysfunctional. Thus, RNA clearance and chromatin remodelling should be considered complimentary processes rather than sequentially different entities [31].

A collection of factors newly identified as licensors and specifiers regulates the transcriptional apparatus itself. Licensors provide regulations that facilitate transcriptional initiation by governing chromatin accessibility, nuclear architecture, and the functionality of the transcriptional apparatus [76]. Specifiers regulate the activation of several gene sets required for distinct embryonic activities at different developmental stages. This paradigm is beneficial as it demonstrates that ZGA encompasses more than only initiating transcription. In the absence of appropriately timed stage-specific specifiers, a transcriptionally permissive genome may fail to initiate the necessary developmental program [10]. Conversely, possessing transcription factors without appropriate licensing will not circumvent structural repression. The molecular regulation of MZT depends on the combination of permissive architecture and instructive sequence-specific control. In embryonic biology, licensors initiate transcription, while specifiers determine the transcriptional content. Arrest phenotypes may arise from the breakdown of any stage, resulting in mechanistically diverse ZGA failure despite morphological similarities [41].

Regulators of heterochromatin and chromatin-state transitions that provide extensive genomic organization also contribute to nuclear reprogramming during MZT. Investigations in vertebrate models have shown that MZT is linked to the formation of genome-wide heterochromatin, dependent on zygotic transcription and the degradation of maternal RNA [77]. These discoveries are fundamentally important as they illustrate that transcript turnover serves both as a cytoplasmic housekeeping mechanism and as a factor influencing nuclear architecture. Eliminating certain maternal transcripts may alleviate obstacles to chromatin compaction pathways, facilitating the emergence of a more developmentally suitable epigenomic environment [78]. An example of a mechanism is the regulation of chromatin remodelers, which may postpone the establishment of heterochromatin and destabilise nuclear structure. This rearrangement is crucial for the early embryo, since transcription must occur inside a genome that is both accessible and partitioned into functional domains. Excessive openness, persistent chromatin states in the mother, or inadequate compartmentalisation can all diminish transcriptional accuracy. MZT involves both gene activation and the systematic rearrangement of the genome as a transcriptional substrate [79].

A more thorough systems-level perspective is obtained by examining the RNA-bound proteome, which undergoes significant alterations throughout the MZT. RNA-binding proteins operate collaboratively within a dynamic regulatory network that evolves from pre-ZGA to post-ZGA stages of development [80]. Substantial modifications in RNA interactomes indicate that embryogenesis is linked to the reorganisation of proteins permitted to bind and interpret maternal transcripts. This flexibility indicates that the regulation of RNA destiny occurs in phases throughout development, with distinct groups of RBPs responsible before and after genome activation. A notable conclusion is that embryo arrest may signify a disruption in network coordination rather than a mutation or lack in a unique component. If RNA-binding landscapes fail to transition appropriately, the embryo may persist in an oocyte-like post-transcriptional state or begin embryonic transcription without enough clearing of inherited regulatory signals. This kind of systemic failure elucidates why early developmental stoppage often seems to result from several factors, rather than being only attributable to cellular morphology or conventional karyotypic analysis [81].

A notable emerging regulator is the autophagy-associated LC3B-mediated degradation of maternal mRNA. Autophagy has traditionally been seen as a process for the recycling of proteins and organelles. It now seems to have a direct function in RNA control during MZT. Researchers have identified LC3B as an RNA-binding protein that facilitates the clearance of maternal mRNA [82]. Its kinetics may surpass those of the traditional BTG4-CCR4-NOT route for some targets. These results enhance the conceptual framework of MZT regulation by linking transcript turnover to cytoplasmic quality-control systems and cellular metabolic conditions. The functional ramifications are substantial. Cytoplasmic homeostasis, organelle turnover, and RNA clearance may be interrelated processes rather than independent ones. Metabolic dysfunction or insufficient autophagy may impact both transcript remodelling and cellular energetics. The developmental halt after autophagy suppression reinforces the idea that a successful shift to embryonic regulation requires not only effective RNA-binding and deadenylation processes but also an operational cytoplasmic recycling system. This integration may be particularly relevant in human infertility, as oocyte quality and metabolic competency can affect embryo viability [83].

## 6. Disruption of the Maternal-Zygotic Transition and Embryo Development

The inadequacy of the maternal–zygotic transition is a prominent biological cause for early embryonic arrest in human IVF. Fertilization may occur properly, pronuclear formation may be completed, and initial cleavage divisions may take place; nevertheless, development often halts between the zygote and the 8-cell stage. These findings indicate that transitioning from maternal regulation to embryonic transcriptional control is impossible. In this instance, developmental arrest is not a fortuitous occurrence. Embryology studies suggest that a significant proportion of developmental failures in IVF transpire during the cleavage phases, particularly during the maternal-zygotic transition, specifically between the zygote and 8-cell stages; however, precise figures remain undetermined. Conversely, developmental cessation after successful big ZGA and compaction is more likely indicative of abnormalities associated with subsequent embryonic differentiation, metabolic dysfunction, aneuploidy, or blastocyst maturation. It is attributable to issues with the timing of transcript clearance, translational activation, and genome activation. Molecular data increasingly suggests that early embryo failure is often due to processes happening before or during maternal-zygotic transition, rather than after blastocyst development. Distinct patterns of early developmental arrest can be mechanistically interpreted based on disruption of specific components of the maternal-zygotic transition, as summarised in Table 2.

Table 2 links specific molecular defects of the MZT with characteristic patterns of embryonic arrest. Different stages of developmental failure may reflect disruption of distinct but interconnected regulatory processes.

A prominent failure in MZT occurs when maternal mRNA clearance is ineffective. Prolonged presence of maternal transcripts throughout development inhibits the activation of the embryonic genome. Accumulated transcripts may continue to regulate oocyte-specific programs or interfere with transcriptional networks critical for early development [83]. Research on human embryos indicates that those arrested at the zygote or early cleavage stages often exhibit aberrant retention of maternal mRNAs, along with reduced expression of proteins that promote transcript destruction. Diverse phenotypic patterns have been delineated. Early arrest correlates with issues in maternal-driven decay pathways, while later arrest is associated with difficulties in zygotic-driven clearance. The results indicate that the timing and thoroughness of transcript removal directly influence developmental progression [84].

Another significant manner in which developmental failure may occur is via the disruption of translational control. Inadequate regulation of translation renders maternal transcripts insufficient. Ribosomes must selectively include certain transcripts to synthesise proteins that facilitate chromatin remodelling, cell cycle progression, and transcriptional competence [85]. Despite the constant amount of transcripts, inadequate translation of crucial maternal factors results in a functional deficiency. Conversely, excessive or insufficient translation may lead to the accumulation of unnecessary proteins, so further destabilizing the developmental program. Experimental investigations and translational profiling in model systems indicate that changes in translational dynamics and selective protein synthesis during early embryogenesis may correlate with diminished developmental potential. Nonetheless, direct ribosome profiling data from human preimplantation embryos with specified developmental outcomes are few [86].

Issues with chromatin remodeling exacerbate the difficulty of transitioning to embryonic regulation. Genome activation necessitates that chromatin be in a conformation conducive to the access of transcription factors and RNA polymerase II to regulatory areas [87]. The incomplete reprogramming of paternal or maternal chromatin, the existence of restrictive histone modifications, or the inability to establish the appropriate chromatin architecture may impede transcriptional activation. Embryos exhibiting delayed or incomplete chromatin remodeling often fail to initiate or sustain zygotic gene activation, hence hindering their progression through the first phases of cleavage. Chromatin remodeling largely depends on proteins synthesized from maternal transcripts. Hence, deficits in translation and RNA clearance may accidentally impede nuclear reprogramming, underscoring the interconnected elements of MZT control [88].

Epigenetic dysregulation is an additional factor contributing to the failure of MZT. To create a transcribable genome, the dynamics of DNA methylation and histone modifications must be meticulously regulated [52]. Abnormal methylation patterns, failure to eliminate repressive marks, or retention of epigenetic signals from gametes when they should not persist may all inhibit gene activation. These abnormalities may lead to anomalous transcriptional patterns, altered developmental timing, and reduced embryo viability. Epigenetic instability during early development may have lasting consequences that extend beyond preimplantation stages, affecting implantation potential and foetal development. In the context of MZT, epigenetic errors often exacerbate developmental issues by interfering with RNA regulation and translation [89].

Metabolic and cytoplasmic variables also affect vulnerability to MZT failure. Mitochondrial malfunction, alterations in ATP synthesis, and oxidative stress may disrupt processes such as translation, RNA turnover, and chromatin remodelling. During the maternal period, energy metabolism is particularly susceptible since early embryos rely only on mitochondria derived from oocytes [89]. Insufficient energy may hinder cellular protein synthesis and disrupt cytoplasmic structure. Excessive oxidative stress may damage RNA, proteins, and DNA. Autophagy-related mechanisms, which facilitate the recycling of organelles and the clearance of RNA, represent another vulnerability. Compromised autophagic function has been associated with delayed degradation of maternal mRNA and defective genome activation, so establishing a direct relationship between metabolic control and MZT efficacy [90].

Single-cell transcriptomic analyses of human embryos have provided enhanced insights into the molecular dimensions of developmental halt. Embryos that fail to develop usually have transcriptional patterns characterised by an inadequate shift from maternal to embryonic gene expression [91]. Residual maternal transcripts persist, with insufficient activation of embryonic gene networks, resulting in a hybrid state that hinders continued development. In some cases, the activation of certain gene modules is either postponed or absent, indicating a failure of coordinated transcriptional processes. These transcriptome fingerprints support the notion that embryo arrest results from a break in a meticulously organised developmental process rather than an arbitrary failure [92].

The clinical importance of MZT disruption is especially evident in cases of unexplained IVF failure. Cycles with normal fertilization rates but suboptimal embryo development may reveal no identifiable explanations when assessed using conventional criteria like as morphology, sperm quality, or chromosomal integrity. Molecular aberrations affecting maternal transcript control, translation, or genome activation provide a valid explanation for these occurrences. The recognition of MZT failure as a critical mechanism alterations focus from successful fertilization to early developmental competence, underscoring the importance of oocyte-derived variables in determining outcomes.

From a biological perspective, MZT disruption constitutes a systemic failure rather than a localized one. A deficiency at one regulatory level often propagates to several additional pathways. Insufficient transcript clearance alters translation processes, poor translation complicates chromatin remodelling, inadequate chromatin remodelling impedes genome activation and failure to activate the genome exacerbates developmental halt. The cascading consequences clarify the reasons for the severe and permanent character of early embryo failure after a disruption in the transition.

## 7. Clinical Implications in IVF

For an extended period, fertilization rates and morphological grading have been the primary methods to assess embryo viability in assisted reproduction. The existence of two pronuclei and first cleavage divisions are often considered signs of normal development. Nonetheless, a considerable proportion of embryos meeting these characteristics fail to progress to the blastocyst stage or accomplish implantation [93]. This disparity between fertilization and developmental results highlights a shortcoming in current assessment methods and reveals molecular factors that typical assessments neglect. The maternal-zygotic transition provides a physiologically consistent framework for understanding this gap, since it governs the early phases of embryonic competence after fertilization. The clinical implications of MZT extend beyond mechanistic understanding and directly impact embryo assessment and IVF strategy, as outlined in Table 3.

Table 3 outlines the translational implications of MZT dysfunction in IVF. Current clinical approaches are contrasted with emerging perspectives based on molecular understanding of early embryo development.

A prevalent clinical scenario involves normal fertilization followed by early developmental arrest. In some cases, fertilization seems technically effective. Embryonic development ceases at cleavage phases without clear morphological signs. Molecular investigations suggest that these patterns may imply impaired maternal mRNA clearance, defective translational control, or inadequate activation of the embryonic genome [58]. Disruptions in any of these processes may not affect pronuclear formation or the first mitotic divisions, but they might significantly impede future development. Recognizing this pattern transforms the understanding of IVF results from simple fertilization to the quality of molecular programming acquired from oocytes [94].

Maternal variables seem to be the most significant determinants influencing MZT effectiveness and, therefore, embryo competence. The quality of oocytes, formed during folliculogenesis, influences the composition of stored transcripts, the efficacy of translational machinery, and the functionality of mitochondria [95]. Advanced maternal age, metabolic disorders, and alterations in follicular environment have all been associated with diminished developmental potential, despite normal fertilization. These connections correspond with the idea that deficiencies in cytoplasmic maturation and molecular preparedness of the oocyte may impede the advancement to embryonic regulation. Paternal inputs, although essential for genomic integrity, seem to have a less direct influence on early transcript dynamics, highlighting the critical role of the oocyte in first developmental competence [96].

The limitations of morphology-based embryo selection are especially evident when assessed in the context of MZT. Morphological evaluation reveals structural properties and cleavage patterns. It does not provide information about transcript turnover, translational activity, or genome activation [97]. Embryos with similar appearance may have significant molecular differences, leading to varied developmental paths. Time-lapse imaging has improved the evaluation of cleavage kinetics; yet, morphokinetic parameters still do not fully clarify the molecular mechanisms that regulate competence. The integration of molecular insights into embryo evaluation remains limited, highlighting the need for approaches that go beyond visual assessment [98].

Advancements in transcriptome and proteomic technologies have begun to reveal potential biomarkers associated with embryo competency. Analysing gene expression patterns, RNA levels, and metabolic markers provides a clearer representation of the embryo’s molecular condition [99]. Non-invasive techniques, such as the examination of discarded culture medium, have been explored to get molecular data without compromising embryo viability. Preliminary findings indicate that RNA release patterns and metabolic activity may be associated with processes such as the degradation of maternal mRNA and the activation of the genome, despite ongoing study. These approaches must undergo testing, standardization, and validation for their efficacy in predictive applications [100].

Eventually, several molecular evaluation techniques may facilitate the practical assessment of MZT-related embryo viability in IVF facilities. A very promising method for the non-invasive examination of wasted embryo culture medium involves the detection of extracellular RNAs, secreted proteins, metabolites, and cell-free nucleic acids, eliminating the need for direct embryo biopsy. The advancement of single-cell transcriptomic and epigenomic technology has enabled more thorough characterizations of early embryonic gene expression dynamics and zygotic genome activation patterns. Additional improvement in identifying embryos with sufficient maternal transcript clearance and developmental competence may be attained by approaches like as small RNA profiling, proteomic analysis, metabolomics, and integrated multi-omics platforms. Although many of these technologies remain exploratory and encounter significant obstacles regarding standardization, cost, and clinical repeatability, they provide viable technological pathways for future genetic embryo screening beyond traditional morphology-based evaluation.

A more sophisticated understanding of MZT also influences patient counselling and treatment strategies. Cases characterised by recurrent early embryo arrest may benefit from evaluating oocyte-related variables instead of focusing only on fertilization procedures [58]. In some therapeutic scenarios, it may be advantageous to modify stimulation procedures, enhance metabolic conditions, or consider oocyte donation. Comprehending MZT-related failures may also aid in halting recurring cycles that persistently fail due to same causes. This approach emphasises individualised therapy based on biological characteristics rather than using uniform protocols to all patients [101].

Emerging technologies such as single-cell transcriptomics and multi-omics integration may enhance our comprehension of the molecular foundations of embryo competency. Integrating genetic, transcriptomic, and metabolic data may elucidate patterns associated with a successful transition to embryonic regulation [102]. Machine learning approaches used to these datasets may enhance prediction capacities and enable the development of customised embryo selection procedures. Clinical application remains nascent, continuous advancements indicate that molecular profiling might eventually enhance or supplement existing selection methodologies.

Incorporating molecular insights into IVF therapy requires careful assessment of both benefits and limitations. Data derived from experimental systems and limited human investigations need meticulous translation into clinical decision-making. Variations across patients, embryos, and laboratory circumstances complicate the comprehension of molecular discoveries. Despite these challenges, comprehending the maternal-zygotic transition as a crucial element in embryo competence establishes a link between developmental biology and therapeutic practice. This combination might ultimately provide a clearer understanding of IVF outcomes and foster the development of more precise and effective reproductive techniques.

The translational significance of MZT research in assisted reproduction may be enhanced by upcoming technology. The integration of artificial intelligence into single-cell transcriptomics, epigenomics, proteomics, and metabolomics may facilitate the precise identification of embryos exhibiting effective maternal-embryonic transition programs. AI-driven analysis of multi-omics datasets may assist in discovering nuanced molecular signals linked to deficient transcript clearance, improper genome activation, or irregular developmental timing that traditional morphology-based assessments could overlook. Simultaneously, there has been an increasing interest in potential therapies aimed at the embryonic microenvironment during the peri-fertilization phase. Indirectly improving MZT efficiency and embryonic developmental competence may be achieved by optimization of culture medium composition, metabolic support, oxidative stress management, and supplementing techniques that seek to increase cytoplasmic and ooplasmic maturation. While currently mostly experimental, these methodologies indicate the possibility for future IVF techniques that will integrate molecular embryo characterization with tailored therapies for effective maternal-embryonic reprogramming.

## 8. Materials and Methods

A narrative methodology was employed to consolidate existing knowledge regarding the maternal-zygotic transition and its significance to embryo competence in assisted reproduction. The goal was to combine mechanistic insights from developmental biology with clinically relevant findings from human embryology and IVF research. The focus was on molecular pathways that control transcript regulation, translation, chromatin remodelling, and genome activation.

We used PubMed/MEDLINE, Scopus, and Embase to do a structured search of the literature to find studies that were published up to 2026. Search terms encompassed combinations of: “maternal-to-zygotic transition,” “zygotic genome activation,” “maternal mRNA decay,” “M-decay,” “Z-decay,” “oocyte competence,” “early embryo development,” “preimplantation embryo,” and “in vitro fertilization.” We added more terms that were related to specific molecular pathways, such as “translation regulation,” “RNA-binding proteins,” “epigenetic reprogramming,” and “chromatin remodelling.” We also looked through the reference lists of the articles we chose to find more relevant publications.

Studies published in high-impact journals, such as Nature, Cell, Science, Nature Reviews, Nature Communications, Science Advances, and leading reproductive medicine journals like Human Reproduction, Human Reproduction Update, and Fertility and Sterility, were prioritised. Both experimental and clinical studies were evaluated to offer a thorough overview of molecular mechanisms and their translational significance.

The following were included:

(i) studies examining the molecular mechanisms of the maternal-zygotic transition, (ii) investigations into maternal mRNA regulation, translational control, or chromatin remodelling in early embryos, (iii) research involving human preimplantation embryos or clinically relevant models, and (iv) recent reviews offering comprehensive perspectives on early embryogenesis.

Exclusion criteria encompassed studies not pertinent to early embryonic development, reports devoid of mechanistic insight, and publications concentrating solely on advanced stages of development beyond the preimplantation phase.

Because human embryonic material is hard to come by, mechanistic insights from mammalian models, especially mouse studies, were used when they were directly related to biological processes that are the same in all species. We used comparative interpretation carefully and focused on data that was backed up by human studies whenever possible. The data extraction focused on important molecular pathways, regulatory factors, and experimental results that had to do with maternal transcript dynamics, translational regulation, and genome activation. We chose studies based on how relevant they were, how well they were done, and how much they added to our understanding of embryo competence. The synthesis sought to establish a cohesive mechanistic framework rather than a mere quantitative compilation of results, in alignment with the narrative review design. There were no formal risk-of-bias assessment or meta-analytic methods used because the goal was to combine existing evidence into a single idea, not to compare outcomes statistically.

## 9. Discussion 

The initial development of embryos seems to be more significantly affected by the ability to execute a meticulously coordinated molecular transition than by fertilization alone. The interaction between maternal transcript clearance, selective translation, chromatin remodeling, and transcriptional activation determines whether cleavage-stage embryos attain developmental autonomy or experience permanent arrest. Evidence from both experimental and human systems suggests a paradigm in which the failure of temporal alignment between these processes, rather than the lack of individual components, accounts for a substantial part of early developmental failure in IVF. In this context, embryo competence denotes not only the presence of molecular elements but also their precise control about timing, spatial orientation, and sequence.

Kojima et al. (2025),define MZT as a dual reprogramming process that encompasses both cytoplasmic and nuclear compartments, emphasizing that chromatin accessibility is affected by preceding cytoplasmic regulation [4]. Conversely, Bashirullah et al. (2013) emphasize the importance of coordinated maternal RNA degradation and embryonic transcription, suggesting that developmental advancement depends on the concurrent cessation of the maternal program and the initiation of a novel transcriptional network [103]. Yang et al. (2020) further on this concept by demonstrating that the degradation of maternal mRNA is a selective and hierarchical process, whereby various subsets of transcripts undergo distinct processing according to their temporal requirements [70]. This aligns with the findings of Zhang et al. (2024), which demonstrate that selective translation of maternal mRNAs produces proteins essential for chromatin remodeling and transcriptional competence [84]. Liu et al. (2018), enhance comprehension by identifying RNA degradation mechanisms reliant on autophagy [41].

Sha et al. (2026), provide direct human data demonstrating that deficits in both M-decay and Z-decay are substantially associated with embryo arrest [30]. This demonstrates that the persistence of maternal transcripts correlates with the inability to start embryonic transcriptional programs, a finding consistent with Yang et al. (2020), who clarify the impact of inadequate RNA degradation on developmental advancement [70]. Liu et al. (2018), reinforce this association by showing that delayed transcript clearance from impaired autophagy impairs ZGA, suggesting that several molecular mechanisms converge on a common phenotype of developmental halt [41]. Kojima et al. (2025), contextualize these data by proposing that the survival of maternal transcripts alters the nuclear environment, hence indirectly impeding transcriptional activation [4]. Zou et al. (2024), reinforce this view by introducing the concept of transcriptional licensing, proposing that genome activation depends on the establishment of permissive circumstances that may be obstructed by insufficient cytoplasmic reprogramming [10]. Sha et al. (2026), demonstrate that embryos with deficient RNA clearance have reduced expression of ZGA-related regulators, so reinforcing a reciprocal link between transcript degradation and transcriptional activation [30].

Zhang et al. (2024), demonstrate that the translational activation of certain maternal transcripts is essential for chromatin remodeling and ZGA [84]. This indicates that protein synthesis serves as a functional connection between RNA clearance and transcriptional start. Xue et al. (2013), Yan et al. (2017), and Petropoulos et al. (2016) demonstrate that human embryo development adheres to highly organized transcriptional pathways, indicating that gene activation occurs not gradually, but via distinct stage-specific programs [11,13,74]. The disruption of these trajectories, particularly the failure to transition from maternal to embryonic gene expression, illustrates the arrest phenotypes described by Sha et al. (2026), so reinforcing the notion that translational and transcriptional processes are interrelated [30]. Yang et al. (2020), demonstrate that translation and degradation operate simultaneously, with the destiny of transcripts influencing the availability of proteins essential for subsequent regulatory mechanisms, suggesting that an imbalance in RNA management may simultaneously impede both translation and transcription [70]. Zhang et al. (2024), identify certain maternal factors whose translation is essential for chromatin accessibility, indicating that translational failure may directly precede impaired genome activation rather than serving as a subsequent consequence [84].

Laue et al. (2019), demonstrate that MZT is associated with the genome-wide establishment of heterochromatin, therefore linking RNA clearance to the construction of higher-order chromatin [77]. Sysoev et al. (2016), demonstrate that RNA-binding protein networks experience significant alterations throughout the transition, indicating that RNA destiny is governed by dynamic fluctuations in protein-RNA interactions rather than by static regulatory mechanisms [104]. Kojima et al. (2025), synthesize these findings by proposing that cytoplasmic and nuclear reprogramming occur simultaneously, so reinforcing the idea that MZT represents a systems-level change marked by the coordinated reconfiguration of several regulatory levels [4]. Zou et al. (2024), improve this model by distinguishing transcriptional licensors from specifiers, proposing that genome activation depends on both favourable structural circumstances and targeted gene activation [10]. Sysoev et al. (2016), suggest that alterations in RNA-binding protein composition may modify the regulatory framework of the embryo, leading to incorrect stabilization or destruction of transcripts and resulting in developmental failure due to network-level disruption rather than individual factor deficits [104].

Fan et al. (2024), provide cross-species single-cell evidence demonstrating the conservation of core regulatory pathways during MZT, while also identifying species-specific variations in transcription factor networks and metabolic activity, thereby supporting the notion that fundamental mechanisms are preserved but context-dependent [105]. Tesarik et al. (2022), emphasizes the need of care when drawing conclusions from model systems, particularly regarding the timing and regulatory complexities in human embryos [92]. Wei et al. (2026), incorporate these mechanistic insights into a clinical framework by associating maternal molecular factors, including transcript regulation, metabolism, and organelle function, with embryo quality and developmental outcomes, suggesting that early molecular events have direct translational relevance [3]. Sha et al. (2020), further validate this link by demonstrating that compromised maternal mRNA clearance is evident in human embryos with developmental halt, so establishing a direct correlation between molecular malfunction and clinical phenotype [33]. Petropoulos et al. (2016), and Yan et al. (2013), augment these results by illustrating that human embryos follow distinct transcriptional patterns, indicating that aberrations from these programs may act as early markers of impaired developmental competence [13].

Kojima et al. (2025), assert that transitory totipotency during MZT depends on the synchronized control of transcription, translation, and RNA degradation, indicating that a failure to achieve this coordinated condition may impede the establishment of a viable embryonic program [4]. Yang et al. (2020), underscore that the selective safeguarding and translation of certain maternal transcripts are as vital as their degradation, suggesting that both the elimination and preservation of transcripts might be harmful [70]. Liu et al. (2018), enhance this perspective by linking autophagic activity to RNA turnover, indicating that metabolic status and cytoplasmic quality control influence transcript dynamics and developmental progression [41]. Zhang et al. (2024), emphasize the importance of translational control by demonstrating that the activation of certain maternal proteins is crucial for chromatin reprogramming, indicating that deficits in protein synthesis may result in nuclear dysfunction [84]. Zou et al. (2024), emphasize that transcriptional activity requires both permissive and instructional inputs. Issues at any level may result in inadequate or erroneous gene expression [10].

Our investigation supports a hypothesis in which MZT functions as a highly integrated regulatory network, rather than a linear sequence of events. The regulation of RNA degradation, translation, chromatin remodeling, and transcriptional activation defines a limited developmental period in which embryonic competence is formed. Any degree of disturbance may propagate across the network, resulting in an accumulation of issues that manifest as early developmental stagnation. This cascade model explains why embryos with normal fertilization may fail to progress and why morphological assessment often does not reliably predict developmental outcomes. The recognition of MZT as a crucial biological checkpoint redefines early embryo failure as stemming from regulatory misalignment at multiple molecular levels, providing a mechanistic rationale for unexplained IVF failure and emphasizing the need for molecular techniques in embryo assessment.

## 10. Conclusions

The initial development of an embryo relies on a successful transition from maternal to embryonic regulation, rather than only on fertilization. The interaction between maternal mRNA degradation, selective translation, chromatin remodelling, and genome activation determines the progression or halt of development in its early stages. Accumulated data supports a paradigm in which early developmental failure indicates the disintegration of a cohesive molecular program. Factors produced from oocytes define the first regulatory environment, and their rapid removal, together with the initiation of embryonic transcription, provides developmental competence. This approach explains why standard fertilization in IVF does not guarantee embryo development. Emphasizing molecular competency above mere morphology may enhance embryo assessment and therapeutic outcomes

## Figures and Tables

**Table 1:** Core molecular events of the maternal-zygotic transition and their functional roles in early embryogenesis.

Biological Process	Key Molecular Mechanisms	Principal Regulators/Genes	Main Developmental Stage	Functional Role in Early Embryogenesis	Consequences of Dysregulation
Maternal mRNA clearance	Deadenylation, uridylation, transcript decay	BTG4, CCR4-NOT complex, TUT4/7	Oocyte maturation and early cleavage stages	Elimination of oocyte-derived maternal transcripts	Persistence of maternal program, impaired developmental transition, embryonic arrest
Translational control	Selective ribosome recruitment, poly(A) tail regulation	eIF4E1B, CPEB1, DAZL	Pre-ZGA and early cleavage stages	Controlled protein synthesis required for chromatin remodeling and ZGA	Defective protein production, impaired developmental competence
Autophagy-mediated RNA turnover	LC3B-associated RNA degradation and cytoplasmic recycling	LC3B, autophagy-related machinery	Early cleavage stages	Acceleration of maternal transcript clearance and cytoplasmic remodeling	Delayed RNA clearance, defective ZGA, developmental arrest
Chromatin remodeling	Histone replacement, nucleosome repositioning, chromatin opening	SMARCD2, histone modifiers	Pre-ZGA transition	Establishment of transcriptionally permissive chromatin architecture	Reduced chromatin accessibility, transcriptional failure
ZGA	Transcription initiation, RNA polymerase II activation	Transcription factors, transcriptional licensors/specifiers	4–8-cell stage in humans	Acquisition of embryonic transcriptional autonomy	Cleavage-stage arrest and failed embryonic progression
Epigenetic reprogramming	DNA demethylation, histone modification dynamics	TET enzymes, histone-modifying proteins	MZT and early embryogenesis	Establishment of totipotency and developmental plasticity	Aberrant gene expression, reduced embryo viability

**Table 2:** Molecular mechanisms associated with early embryonic arrest in IVF.

Stage of Arrest	Underlying Defect	Molecular Basis	Observed Phenotype	Clinical Correlate
Zygote stage	Impaired M-decay	Failure of maternal RNA clearance	Persistence of maternal transcripts	Fertilization without cleavage progression
2–4 cell stage	Translational dysregulation	Defective ribosome recruitment	Insufficient protein synthesis	Slow or abnormal cleavage
4–8 cell stage	Failed ZGA	Inadequate transcription activation	Lack of embryonic gene expression	Developmental arrest before compaction
Cleavage stage	Chromatin remodeling defects	Inaccessible chromatin, epigenetic imbalance	Delayed or absent transcription	Poor embryo quality despite normal fertilization
Multistage arrest	Combined defects	RNA + translation + chromatin disruption	Hybrid transcriptional state	Recurrent IVF failure

**Table 3:** Clinical implications of maternal-zygotic transition dysfunction in IVF.

Domain	Current Practice	Limitation	MZT-Based Interpretation	Future Direction
Embryo selection	Morphology, morphokinetics	No molecular insight	Does not assess transcript dynamics or ZGA	Integration of molecular biomarkers
Fertilization assessment	2PN evaluation	Does not predict development	Fertilization ≠ competence	Shift focus to post-fertilization biology
Recurrent IVF failure	Empirical approach	Unexplained cases	Possible MZT failure	Oocyte-centered evaluation
Biomarkers	Limited use	Lack of validation	RNA/protein signatures reflect competence	Non-invasive omics (spent media)
Treatment strategies	Standard protocols	Uniform approach	Molecular heterogeneity ignored	Personalised IVF strategies

## Data Availability

No new data were created or analyzed in this study. Data sharing is not applicable to this article.

## Abbreviations

The following abbreviations are used in this manuscript:

BTG-4	B-cell translocation gene 4
CPEB1	cytoplasmic polyadenylation element-binding protein 1
DAZL	deleted in azoospermia-like
DUX4	double homeobox 4
OBOX	oocyte-specific homeobox
